# Research on the Relative Position Detection Method between Orchard Robots and Fruit Tree Rows

**DOI:** 10.3390/s23218807

**Published:** 2023-10-29

**Authors:** Baoxing Gu, Qin Liu, Yi Gao, Guangzhao Tian, Baohua Zhang, Haiqing Wang, He Li

**Affiliations:** 1 College of Engineering, Nanjing Agricultural University, Nanjing 210031, China; gbx@njau.edu.cn (B.G.); 9203011507@stu.njau.edu.cn (Y.G.); whq_nj@126.com (H.W.); lihe@njau.edu.cn (H.L.); 2 School of Cyber Science and Engineering, Southeast University, Nanjing 210096, China; 230229544@seu.edu.cn; 3 SUNWAY-AI Technology (Changzhou) Co., Ltd., Changzhou 213161, China; 4 College of Artificial Intelligence, Nanjing Agricultural University, Nanjing 210031, China; bhzhang@njau.edu.cn

**Keywords:** orchard robot, autonomous navigation, positional parameters, machine vision, YOLO

## Abstract

The relative position of the orchard robot to the rows of fruit trees is an important parameter for achieving autonomous navigation. The current methods for estimating the position parameters between rows of orchard robots obtain low parameter accuracy. To address this problem, this paper proposes a machine vision-based method for detecting the relative position of orchard robots and fruit tree rows. First, the fruit tree trunk is identified based on the improved YOLOv4 model; second, the camera coordinates of the tree trunk are calculated using the principle of binocular camera triangulation, and the ground projection coordinates of the tree trunk are obtained through coordinate conversion; finally, the midpoints of the projection coordinates of different sides are combined, the navigation path is obtained by linear fitting with the least squares method, and the position parameters of the orchard robot are obtained through calculation. The experimental results show that the average accuracy and average recall rate of the improved YOLOv4 model for fruit tree trunk detection are 5.92% and 7.91% higher, respectively, than those of the original YOLOv4 model. The average errors of heading angle and lateral deviation estimates obtained based on the method in this paper are 0.57° and 0.02 m. The method can accurately calculate heading angle and lateral deviation values at different positions between rows and provide a reference for the autonomous visual navigation of orchard robots.

## 1. Introduction

With the rapid development of sensor technology and computer technology, robotic autonomous navigation technology has been applied in orchards on a large scale [1,2,3]. Autonomous navigation technology is the key technology to realize the intelligence of agricultural equipment in orchards, which is conducive to reducing the work intensity of operators, improving work efficiency, and enhancing the quality of operations [4,5,6].

Autonomous navigation technology mainly perceives the dynamic environment around the vehicle through a variety of sensors to plan and navigate the path and complete operations, such as orchard weeding [7,8], furrowing and fertilizing [9,10,11], and picking [12,13]. Commonly used navigation methods are classified as Global Navigation Satellite Systems (GNSS)/Global Positioning Systems (GPS) [14,15], Light Detection and Ranging (LIDAR) [16,17], vision sensors [18,19], and multi-sensor fusion navigation [20]. Wei Shuang et al. [21] proposed a pure tracking model based on the GNSS autonomous navigation path search method for agricultural machines and pre-sighting point search. In straight-line navigation, the root mean square error of navigation is 3.79, 4.28, and 5.39 cm when the speed is 0.8, 1.0, and 1.2 m/s, respectively. Luo Xiwen et al. [22] developed an automatic navigation control system based on DGPS; the maximum error of this system is less than 15 cm, and the average error is less than 3 cm in straight-line navigation when the tractor is at a speed of 0.8 m/s. However, when the fruit tree branches and leaves are luxuriant, the canopy is closed, and the environment between the rows of the tree is almost in the semi-closed state. Satellite signals are then blocked by the canopy, leading to certain limitations of the positioning and navigation method based on GNSS/GPS positioning and navigation methods subject to certain limitations. Liu Weihong et al. [23] proposed an orchard inter-row navigation method based on 3D LiDAR, the method in the real pear orchard with mobile robot tracking of tree rows at a speed of 1.35 m/s. The maximum lateral deviation obtained is less than 22.1 cm. 3D LiDAR and subject to weather conditions. It is expensive, greatly increasing the cost of inputs in orchard environments, and therefore difficult to use universally. Han Zhenhao et al. [24] proposed an orchard visual navigation path recognition method based on the U-Net network, which uses a camera to collect images and semantically segment the road information in the image to calculate the navigation path. Using this method to drive in the orchard road with a width of about 3.1 m, the average distance error ratio is 1.4%, which is about 4.4 cm, and the average processing time of a single frame of image is 0.154 s.

In recent years, deep learning has made breakthrough progress in image classification research and has also driven the rapid development of target visual detection [25,26,27]. Deep learning-based target detection models include two-stage target detection models and single-stage target detection models [28,29]. Two-stage target detection models have higher detection accuracy but slower detection speed; therefore, most real-time detection tasks currently use single-stage target detection models. Typical single-stage detection models include the YOLO [30,31,32] family of models and the SSD [33] model. Xie Shuang et al. [34] used an improved YOLOv8 model for tea recognition, which combines deformable convolution, an attention mechanism, and improved spatial pyramid pooling, thus enhancing the model’s ability to learn complex target invariance, reducing the interference of irrelevant factors, and achieving multi-feature fusion, which improves the detection accuracy. Wang et al. [35] constructed a model that fuses YOLO v5s and the attention mechanism of convolutional neural network model YOLO_CBAM for the detection of spiny calyx lobelia weed. They devised a method for slicing high-resolution images. This method constructs the dataset by calculating the overlap rate to reduce the possibility of loss of details due to compression of high-resolution images during training, and the final accuracy is 92.72%. Tian et al. [36,37] successfully achieved the detection of grape maturity and weed identification and localization through the improved YOLOv4 algorithm. The experimental results show that this method has high accuracy.

By summarizing the work of relevant researchers, we can find that when working, orchard robots or agricultural robots mostly need navigation satellite systems for absolute positioning. However, in the actual working process, due to the obstruction of the tree canopy, the orchard robot may not be able to receive satellite signals normally. At this time, the orchard robot needs to obtain its relative pose with the tree row through its own sensors in real time. In this way, the orchard robot can navigate autonomously in the tree row.

This paper proposes a method based on a binocular camera to determine the positional parameters of an orchard robot. When the orchard robot advances between rows, the binocular camera acquires images and transmits them to the improved YOLOv4 model for fruit tree trunk detection, obtains the camera coordinates of the trunks, and then obtains the ground projection coordinates of each trunk after coordinate conversion. The ground projection coordinates of different sides of the orchard robot are combined to take the midpoint, the least squares method is used to fit a straight line to the midpoint to obtain the navigation path, and the heading angle and lateral deviation values of the orchard robot are obtained through calculation.

## 2. Materials and Methods

In this paper, the proposed method for estimating the positional parameters between rows of orchard robots consists of four parts: fruit tree trunk detection, fruit tree trunk localization, navigation path calculation, and calculation of positional parameters. The detection of fruit tree trunks is based on the improved YOLOv4 model to detect the trunks on both sides of the orchard robot; the positioning of fruit tree trunks is based on the parallax principle after the binocular camera acquires the image to obtain the camera coordinates of the fruit tree trunks; the calculation of the navigation path is based on the camera coordinates of the fruit tree trunks obtained from the previous step through the coordinate transformation to obtain the ground projection coordinates, the combination of projection coordinates of the different sides of the orchard robot to take the mid-point; and the mid-point is calculated using the least-squares method. The least squares method is used to fit the midpoint to a straight line; the attitude parameters are obtained by calculating the straight line relationship between the fitted straight line and the vehicle base coordinate system.

### 2.1. Hardware Composition

**Figure 1:**
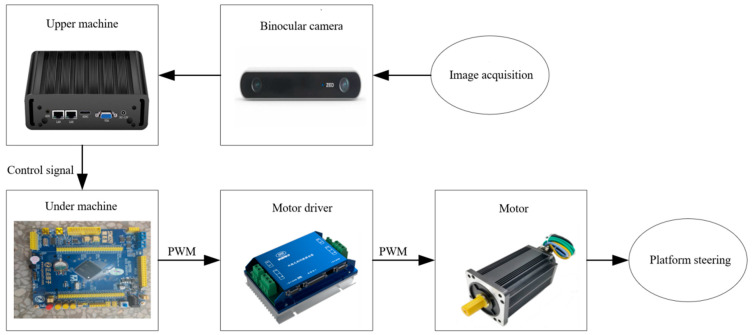
Entire design of the system.

In this study, the electric-driven tracked orchard operation platform designed by the College of Engineering of Nanjing Agricultural University is used as the mobile carrier, which is driven by two 48 V DC servo motors; the motors are driven and controlled by one dual-channel servo motor driver; the upper computer is the New Creation Cloud Embedded Industrial Controller with an external display screen; STM32 is selected as the lower computer to control the servo motor driver of the mobile carrier; the vision sensor uses the ZED2i (polarized version) high-definition camera; the camera’s focal length f is 1.8 mm, the base distance b is 120 mm, and the camera captures the left and right images with a resolution of 1280 × 720. The overall scheme of the system is shown in Figure 1:

### 2.2. Technological Route

**Figure 2:**
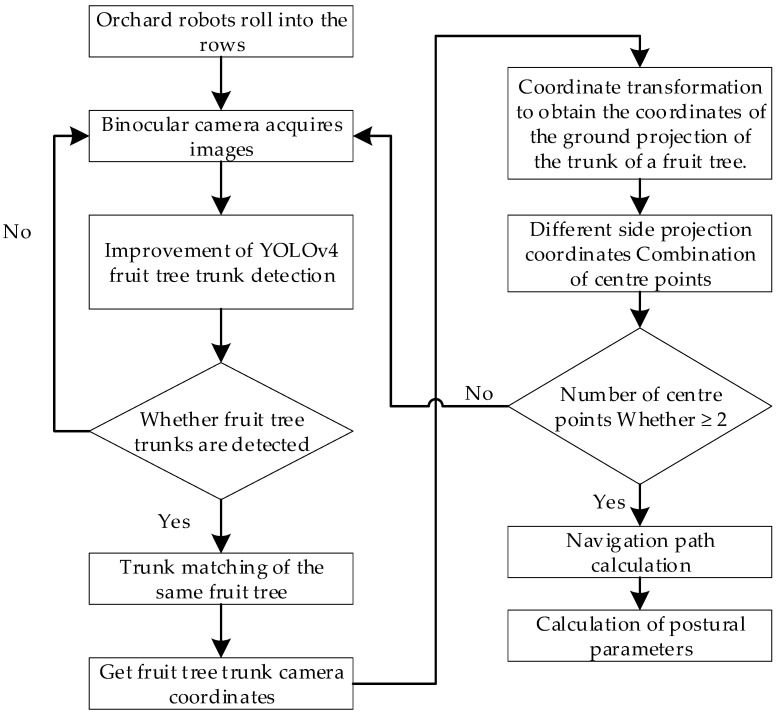
Technical process of estimating pose parameters between trees.

The technical flow of the machine vision-based inter-row position estimation and navigation method for orchard robots at night is shown in Figure 2.

### 2.3. Improvements to the YOLOv4 Algorithm

#### 2.3.1. YOLOv4 Algorithm

The YOLO family of algorithms is one of the most advanced target detection algorithms in the world. The YOLOv4 algorithm uses the CSPDarknet53 as the backbone network for feature extraction. It uses the CBM module (consisting of Convolution, Normalization, and Mish activation functions) and the CBL module (consisting of Convolution, normalization, and LeakyReLU activation functions) for feature extraction. It also joins the Spatial Pyramid Pooling Networks (SPPNet) and Path Aggregation Network (PANet) modules for feature extraction while adding two network modules, SPPNet and PANet, so that the target detection accuracy of the YOLOv4 algorithm is better than that of the YOLOv3 algorithm. Additionally, the YOLOv4 algorithm adds image enhancement during training, thus further expanding the dataset.

#### 2.3.2. Improvement of the YOLOv4 Algorithm

In this paper, the YOLOv4 algorithm is used to detect fruit tree trunks. When the orchard robot travels between rows, the environment is relatively complex, and the detection accuracy of fruit tree trunks is an important prerequisite to ensure that the orchard robot can safely navigate. Therefore, it is necessary to carry out certain optimizations of the YOLOv4 algorithm so as to improve the detection accuracy of fruit tree trunks.

**Figure 3:**
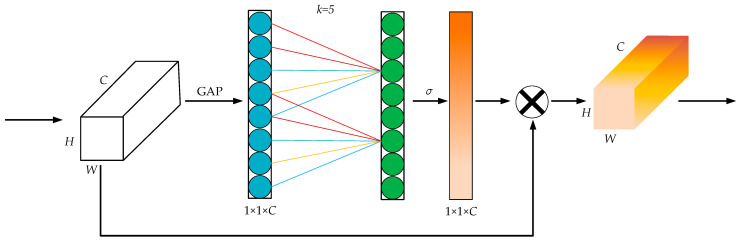
The ECA module. Note: *C*, *W*, and *H* denote the feature channel size, width, and height of the feature map, respectively; *σ* denotes the Sigmoid activation function.

In this paper, we introduce an efficient channel attention mechanism ECA module, as shown in Figure 3, where *k* = 5 is used as an example. The ECA module maintains the dimensionality of the channel, performs a global average pooling (GAP) operation on the channel, and generates the channel weights using a Sigmoid activation function (*σ*) after one-dimensional convolution. Finally, it multiplies the channel weights with the original feature layer one by one to obtain a new feature layer, where the convolution kernel scale *k* can be adaptively determined and is proportional to the channel dimension. Therefore, if the channel dimension *C* is known, then the convolutional kernel scale *k* can be obtained by calculating Equation (1). The ECA module adds little computational effort to the algorithm and improves performance in all aspects.

(1) $$k=\psi (C)={\left|\frac{\log_{2}(C)}{a^{\prime }}+\frac{b^{\prime }}{a^{\prime }}\right|}_{\mathrm{odd}}$$

where |*|_odd_ denotes the nearest odd number to |*|, and *a*′ and *b*′ are function coefficients. In this paper, *a*′ takes the value of 2, and *b*′ takes the value of 1.

**Figure 4:**
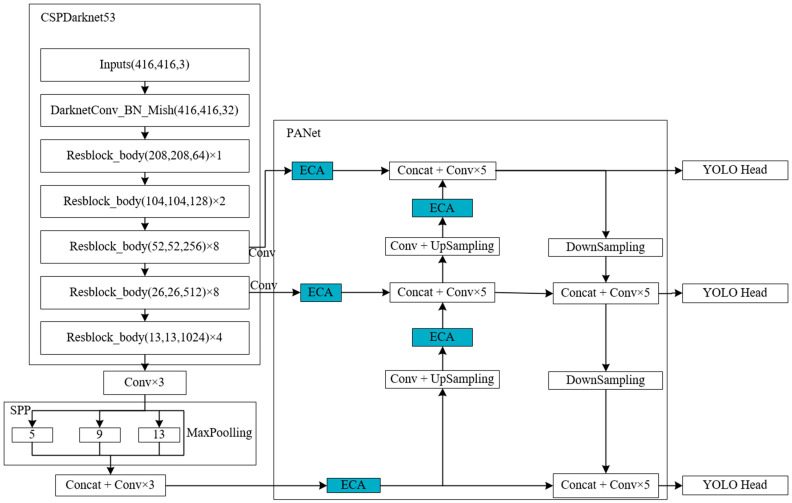
The network model of the ECA5-YOLOv4 algorithm.

In the YOLOv4 algorithm, the input dataset outputs three kinds of feature layers—52 × 52 × 256, 26 × 26 × 512. and 13 × 13 × 1024—after feature extraction by the backbone feature extraction network CSPDarknet53. Here, the 52 × 52 × 256 and 26 × 26 × 512 are input into the PANet module after one convolution, and the 13 × 13 × 1024 feature layer is input into PANet after channel stacking and three convolutions after the SPP module. In this paper, the channel attention mechanism ECA module is added to these three feature layers before channel stacking and five convolution operations in PANet. In the PANet module, there are two up-samplings of the feature layer. This paper adds an ECA module after up-sampling and performs an enhanced feature extraction operation on the feature layer after up-sampling. This paper, therefore, adds five ECA modules to the original YOLOv4 algorithm and obtains the optimized new algorithm ECA5-YOLOv4 algorithm. The network model of the ECA5-YOLOv4 algorithm is shown in Figure 4.

### 2.4. Fruit Tree Trunk Positioning

#### 2.4.1. Fruit Tree Trunk Camera Coordinates

Fruit tree trunks are detected by improving the YOLOv4 model on the left and right images captured by the binocular camera. After detecting the fruit tree trunks, the fruit tree trunks are matched, and when the same fruit tree trunks in the left and right images are matched successfully, the pixel parallax *D* and image parallax *d* of the same fruit tree trunks in the left and right images are obtained; according to the principle of triangulation of the binocular camera, the camera coordinates of the fruit tree trunks are obtained *(X_C_*, *Y_C_*, *Z_C_)*. The method in this paper is to project the ground so the *Y_C_* coordinates do not need to be solved to simplify the calculation process. The pixel coordinates of the geometric center of the fruit tree trunk in the left image are ($u_{L},v_{L}$), and the image coordinates are ($x_{L},y_{L}$); the pixel coordinates of the geometric center of the fruit tree trunk in the right image are ($u_{R},v_{R}$), and the image coordinates are ($x_{R},y_{R}$):

(2) $$D=\left(u_{L}-u_{R}\right)$$

(3) $$d=\left(x_{L}-x_{R}\right)$$

**Figure 5:**
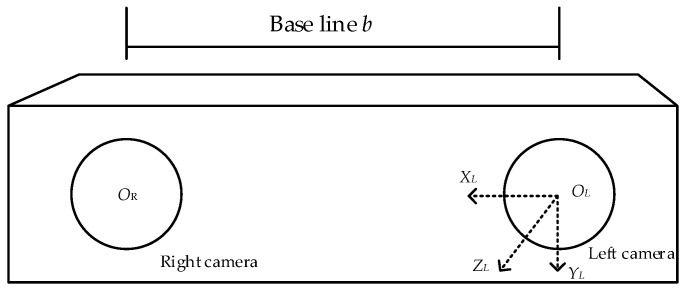
Structure of binocular camera.

Binocular camera left and right camera optical centers were expressed by *O_L_*, *O_R_*, with *O_L_* as the origin, horizontally to the right for the axis *X_L_* positive direction, vertically down for the axis *Y_L_* positive direction, and horizontally forward for the positive direction of the *Z_L_*, to establish the camera coordinate system *O_L_-X_L_Y_L_Z_L_*. f indicates the focal length of the binocular camera in mm; *b* indicates the baseline of the left and right cameras in mm. The structure of the binocular camera is shown in Figure 5.

The principle of conversion of pixel coordinates to image coordinates and the similarity triangle relationship between image coordinates and camera coordinates is rendered as follows:

(4) $$X_{C}=\frac{Z_{C}\cdot \left(u_{L}-u_{0}\right)}{f_{x}}$$

where $f_{x}$ denotes the pixel coordinate system *u*-axis scale factor, respectively, and *u*_0_ denotes the amount of lateral translation of the image coordinate system origin in the pixel coordinate system.

According to the triangulation method,

(5) $$Z_{C}=\frac{f\cdot b}{d}=\frac{f\cdot b}{x_{L}-x_{R}}$$

The horizontal coordinates of the geometric center of the fruit tree trunk in the pixel coordinate system, $u_{L},u_{R}$ in the left and right images, respectively:

(6) $$\{\begin{array}{c} u_{L}=\frac{f\cdot x_{L}}{f_{x}}+u_{0}\\ u_{R}=\frac{f\cdot x_{R}}{f_{x}}+u_{0} \end{array}$$

From Equations (5) and (6), the depth *Z_C_* is obtained as follows

(7) $$Z_{C}=\frac{f_{x}\cdot b}{u_{L}-u_{R}}$$

Substituting Equation (7) into Equation (4), there is:

(8) $$X_{C}=\frac{b\cdot \left(u_{L}-u_{0}\right)}{u_{L}-u_{R}}$$

The coordinates (*Xc*, *Zc*) of the fruit tree trunk in the planar coordinate system *X_L_O_L_Z_L_* are obtained from Equations (7) and (8) where, *b*, *u*_0_, *f_x_* are camera internal parameters, which can be obtained by camera calibration.

#### 2.4.2. Fruit Tree Trunk Coordinate Conversion

**Figure 6:**
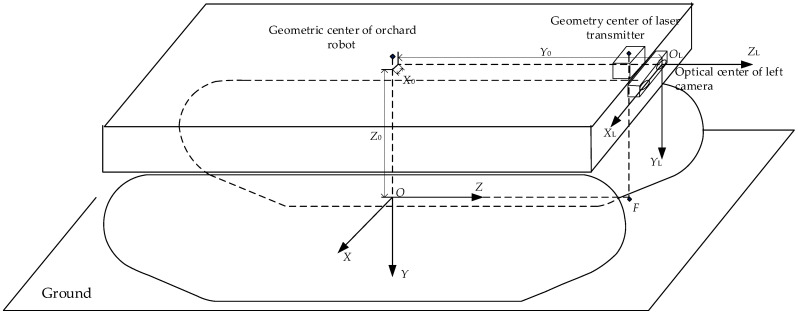
Coordinate system and transformation relations.

Using the coordinate system and conversion relationship shown in Figure 6, this paper will provide the orchard operation platform on the ground projection for the ground coordinate system origin *O*: the binocular camera installed in the front of the vehicle in the center; binocular camera geometric center of the projection of the ground for the point *F*; $\vec{OF}$ for the ground coordinate system axis *Z* positive direction; horizontally to the right for the axis of the X-positive direction; and the establishment of the ground coordinate system *XOZ*. The ground coordinate system can be obtained by the camera coordinate system in the planar coordinate system *X_L_O_L_Z_L_*_._ The translation of *X* and *Z* direction is *X*_0_ and *Z*_0,_ respectively. When the camera coordinates are converted to ground coordinates, the *Y*-axis direction is not to be calculated. The fruit tree trunk in the plane coordinate system *X_L_O_L_Z_L_* coordinates for (*X_C_*,*Z_C_*), and, in the ground, coordinate system coordinates for (*X_g_*,*Z_g_*); the conversion relationship is shown in Equation (9).

(9) $$\{\begin{array}{c} X_{g}=X_{C}-X_{0}\\ Z_{g}=Z_{C}+Z_{0} \end{array}$$

### 2.5. Calculation of Navigation Path and Attitude Parameters

When the orchard robot is traveling between rows, the binocular camera is used to locate the tree trunks. Then, the coordinate conversion is used to obtain the sitting mark of a plurality of fruit tree trunks in the ground coordinate system as *G_i_*(*X_gi_*,*Z_gi_*). Among them, the coordinate point of *G_i_*(*X_gi_*,*Z_gi_*) < 0 is regarded as being located on the left side of the orchard operation vehicle, and at this time, the left-side sitting mark is *G_Lj_*(*X_glj_*,*Z_glj_*). The coordinate point of *X_gi_* ≥ 0 is regarded as being located on the right side of the orchard operation vehicle, and at this time, the right-side sitting mark is *G_Rk_*(*X_grk_*,*Z_grk_*).

The combination of points on the left and the right is taken to be the midpoint, with a total of *j × k* midpoints, denoted *C_j,k_*(*X_j,k_*,*Z_j,k_*).

(10) $$\{\begin{array}{c} X_{j,k}=\frac{X_{glj}+X_{grk}}{2}\\ Z_{j,k}=\frac{Z_{glj}+Z_{grk}}{2} \end{array}$$

#### 2.5.1. Navigation Path Calculation

The obtained *j × k* midpoints are fitted with a straight line using the least squares method, and the calculated straight line is the navigation path, noting that the fitted straight line is *Z = a*_1_*X + a*_0_. The calculation process is as follows:

(11) $$\left[\begin{array}{cc} j\times k & \sum_{j=1,k=1}^{j,k}X_{j,k}\\ \sum_{j=1,k=1}^{j,k}X_{j,k} & \sum_{j=1,k=1}^{j,k}X_{j,k}^{2} \end{array}\right]\cdot \left[\begin{array}{c} a_{0}\\ a_{1} \end{array}\right]=\left[\begin{array}{c} \sum_{j=1,k=1}^{j,k}Z_{j,k}\\ \sum_{j=1,k=1}^{j,k}X_{j,k}Z_{j,k} \end{array}\right]$$

Find *a*_0_ and *a*_1_ from Equation (11):

(12) $$\{\begin{array}{c} a_{0}=\frac{\sum_{j=1,k=1}^{j,k}X_{j,k}^{2}\sum_{j=1,k=1}^{j,k}Z_{j,k}-\sum_{j=1,k=1}^{j,k}X_{j,k}\sum_{j=1,k=1}^{j,k}X_{j,k}Z_{j,k}}{(j\times k)\cdot \sum_{j=1,k=1}^{j,k}X_{j,k}^{2}-{\left(\sum_{j=1,k=1}^{j,k}X_{j,k}\right)}^{2}}\\ a_{1}=\frac{\sum_{j=1,k=1}^{j,k}X_{j,k}Z_{j,k}-\sum_{j=1,k=1}^{j,k}X_{j,k}\sum_{j=1,k=1}^{j,k}Z_{j,k}}{\sum_{j=1,k=1}^{j,k}X_{j,k}^{2}-{\left(\sum_{j=1,k=1}^{j,k}X_{j,k}\right)}^{2}} \end{array}$$

#### 2.5.2. Calculation of Postural Parameters

**Figure 7:**
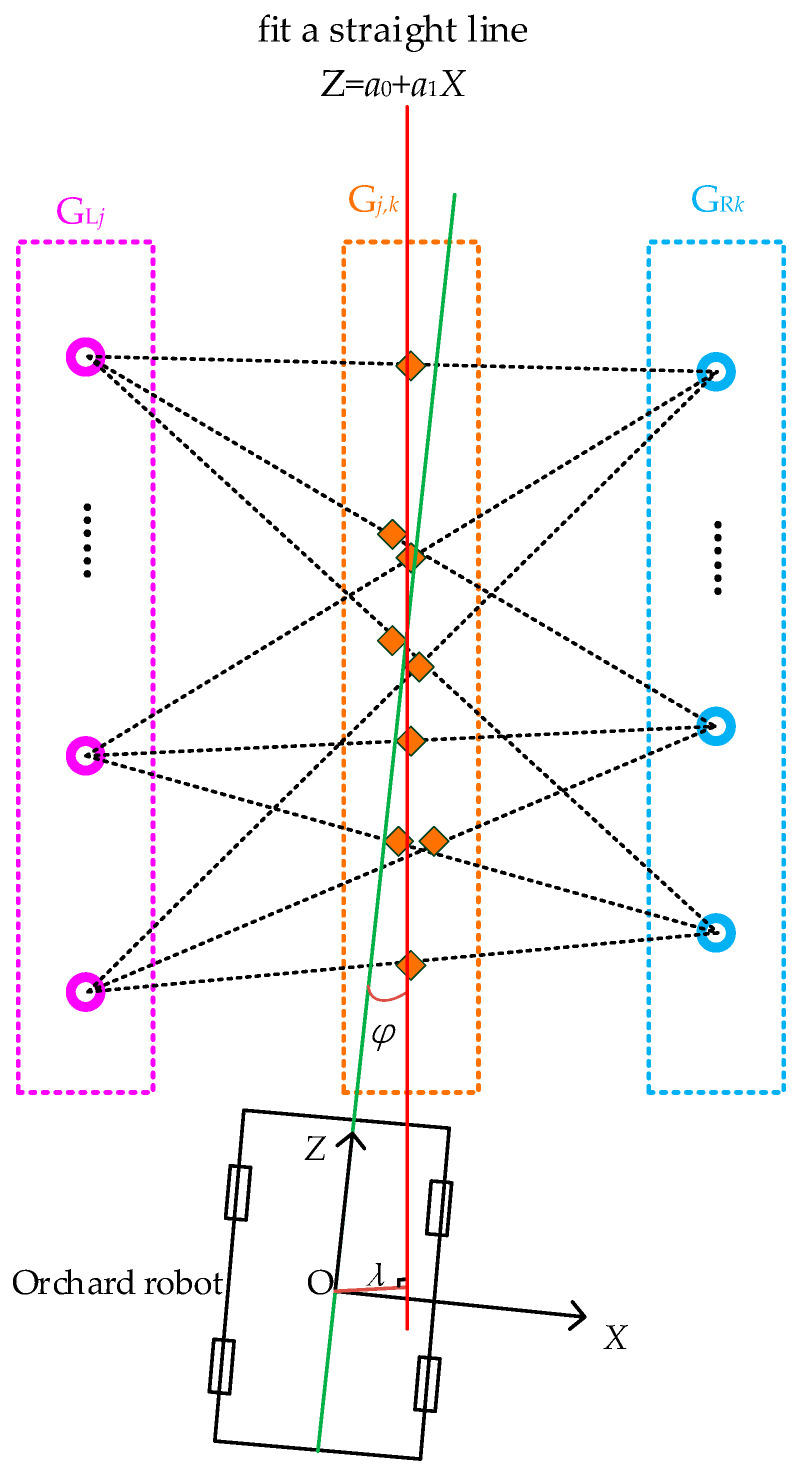
Driving position relationship between rows.

The positional relationship of the orchard operation platform when traveling between rows is shown in Figure 7, and the traveling direction of the vehicle is the same as the positive direction of the *Z*-axis, which can be expressed by *X =* 0. Therefore, the heading angle *φ* of the vehicle can be obtained by calculating the angle between *X =* 0 and *Z = a*_1_*X + a*_0_; the lateral deviation *λ* of the vehicle can be obtained by calculating the perpendicular distance from the origin *O* to the fitted straight line *Z = a*_1_*X + a*_0_. The calculation formulas are shown in (13) and (14):

(13) $$\varphi =90^{\circ }-\left|\arctan a_{1}\right|$$

(14) $$\lambda =\frac{\left|a_{0}\right|}{\sqrt{1+a_{1}^{2}}}$$

## 3. Results

### 3.1. Data Acquisition and Model Training

The experimental dataset collection site was located in the College of Engineering, Nanjing Agricultural University, and 700 fruit tree trunk images were collected. Data enhancement was performed on the images by adding noise and flipping to expand the dataset to 1500 sheets, including 1200 sheets for the training set and 300 sheets for the validation set. The fruit tree trunk part was labeled using the LabelMe tool with the category information “tree.”

**Figure 8:**
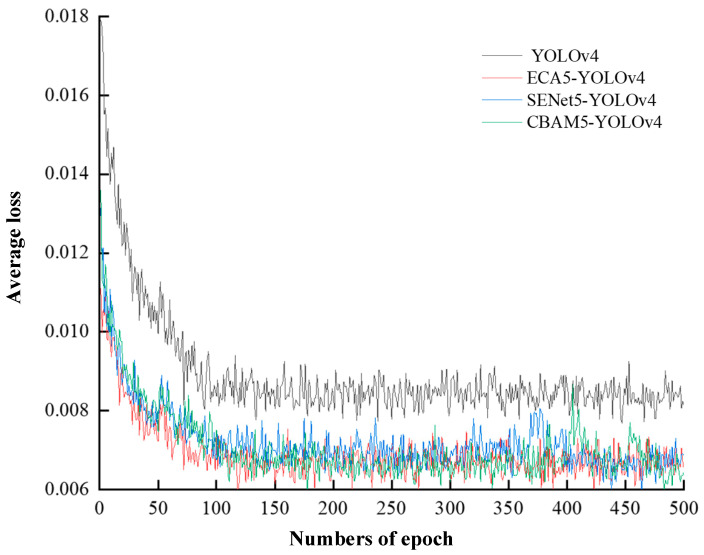
The loss function of model training.

In order to verify the higher accuracy of the ECA5-YOLOv4 algorithm, this paper replaces the five ECA modules in the model with the new attention mechanism modules, SENet module, and CBAM module. The original YOLOv4 algorithm, ECA5-YOLOv4 algorithm, SENet5-YOLOv4 algorithm, and CBAM5-YOLOv4 algorithm were trained for 500 generations using the same dataset, respectively. The loss functions of the training models are shown in Figure 8.

Training platform: Intel(R) Xeon(R) E5 2689 2.60 GHz CPU, 32 G RAM, NVIDIA GeForce GTX 1070 8 G graphics card.

The evaluation metrics of training results in this experiment include Precision (*P*), Recall (*R*), and Frame rate. *P* and *R* are calculated as:

(15) $$P=\frac{T_{P}}{T_{P}+F_{P}}\times 100\%$$

(16) $$R=\frac{T_{P}}{T_{P}+F_{N}}\times 100\%$$

where T_P_ denotes the number of correctly detected fruit tree trunks in the picture, F_P_ denotes the number of detection errors in the picture, and F_N_ denotes the number of missed targets in the picture.

Table 1 shows the training results of the original YOLOv4 algorithm, the ECA5-YOLOv4 algorithm, the SENet5-YOLOv4 algorithm, and the CBAM5-YOLOv4 algorithm.

From the result analysis, the ECA5-YOLOv4 algorithm, SENet5-YOLOv4 algorithm, and CBAM5-YOLOv4 algorithm have shown improvements in precision rate, recall rate, and frame rate compared to the original YOLOv4 algorithm. Among them, the ECA5-YOLOv4 algorithm has the highest precision rate and recall rate of 97.05% and 95.42%, respectively, which are improvements of 5.92%, 2.8%, and 0.22% in precision rate, and 7.91%, 4.41%, and 0.28% in recall rate when compared to the YOLOv4 algorithm, SENet5-YOLOv4 algorithm, and CBAM5-YOLOv4 algorithm, respectively. The SENet5-YOLOv4 algorithm has the highest frame rate, which is 2.56 fps, 0.44 fps, and 0.53 fps higher than the YOLOv4 algorithm, ECA5-YOLOv4 algorithm, and CBAM5-YOLOv4 algorithm, respectively.

### 3.2. Posture Parameter Determination Test

#### 3.2.1. Binocular Camera Internal Reference Measurement

In this experiment, a ZED2i (polarized version) HD camera was used; the focal length f of the binocular camera was 1.8 mm, the base distance b was 120 mm, and the resolution of the camera grabbing the left and right images was 1280 × 720. The internal parameters of the camera were calibrated using the Software Development Kit (SDK 4.0) that comes with the binocular camera. According to the needs of fruit tree trunk localization, u0 and result were obtained as 645.25 pixels and 529.88 pixels, respectively.

#### 3.2.2. Experimental Design and Evaluation Indicators

This experiment was carried out in the College of Engineering of Nanjing Agricultural University, and the orchard robot was driven into the rows of fruit trees and stopped at any position in the rows, at which time the real values of the heading angle and lateral deviation of the orchard robot were measured and recorded as φ_t_, λ_t_; the values measured by the method in this paper are estimated values and recorded as φ_e_, λ_e_. The difference between the real values of the heading angle and lateral deviation and the estimated values are the error values and recorded as E_φ_, E_λ_, which were taken as the evaluation indexes of the present experiment. The valuation index is calculated as follows:

(17) $$E_{\varphi }=\left|\varphi_{t}-\varphi_{e}\right|$$

(18) $$E_{\lambda }=\left|\lambda_{t}-\lambda_{e}\right|$$

#### 3.2.3. Experimental Results and Analysis

**Figure 9:**
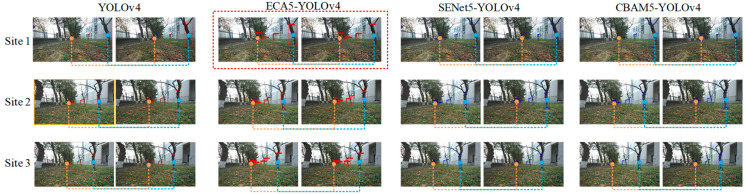
The results of tree trunk detection based on different models.

**Figure 10:**
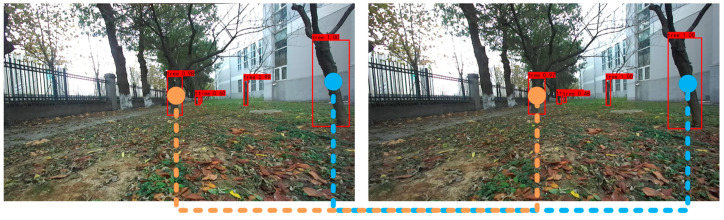
Enlarged ECA5-YOLOv4 image.

The fruit tree trunks are detected by different models, and the positional parameters of the orchard robot are also calculated. The fruit tree trunk results are shown in Figure 9, and the calculation results are shown in Table 2. The ECA5-YOLOv4 image at Site 1 is enlarged separately, and the effect is shown in Figure 10. In Figure 9, the actual heading angle and lateral deviation of the orchard robot from Position 1 to Position 3 are 150.2° and 0.53 m, 158.5° and 0.48 m, and 160.4° and 0.52 m, respectively. From Table 2, it can be seen that the best estimation of the positional parameters of the orchard robot was made by the ECA5-YOLOv4 model, with the mean values of the errors of heading angle and lateral deviation being 0.57° and 0.02 m. The results were compared with those of the original YOLOv4, SENet5-YOLOv4, and CBAM5-YOLOv4 models; the mean values of errors were reduced by 1.50° and 0.01 m, 0.86° and 0.03 m, and 0.60° and 0.03 m, respectively.

## 4. Discussion

In this study, based on the YOLOv4 model, the ECA5-YOLOv4 model is obtained by introducing five ECA attention mechanism modules into the PANet module of the model, and the ECA5-YOLOv4 model can efficiently and accurately detect the trunks of fruit trees, which can provide a guarantee for obtaining the positional parameters of the orchard robot.

(1) Compared with the original YOLOv4, SENet5-YOLOv4, and CBAM5-YOLOv4 models, the accuracy of the ECA5-YOLOv4 model for fruit tree trunks improved by 5.92%, 2.8%, and 0.22%, respectively;

(2) The estimation method of inter-row position parameters of the orchard robot proposed in this paper obtains the mean values of the errors of heading angle and lateral deviation as 0.57° and 0.02 m, with low errors, which can provide a theoretical basis for the orchard robot to navigate between the rows of fruit trees.

## Figures and Tables

**Table 1:** Results of model training.

Model	Precision/%	Recall/%	$\mathrm{Frame}\;\mathrm{Rate}/(\mathrm{Frame}\cdot s^{-1}$)
YOLOv4	91.13	87.51	15.47
ECA5-YOLOv4	97.05	95.42	17.59
SENet5-YOLOv4	94.25	91.01	18.03
CBAM5-YOLOv4	96.83	95.14	17.50

**Table 2:** Test results of pose parameters determination.

Site	YOLOv4		ECA5-YOLOv4		SENet5-YOLOv4		CBAM5-YOLOv4	
	Heading Angle *φ*_e_/°	Lateral Deviation *λ*_e_/m	Heading Angle *φ*_e_/°	Lateral Deviation *λ*_e_/m	Heading Angle *φ*_e_/°	Lateral Deviation *λ*_e_/m	Heading Angle *φ*_e_/°	Lateral Deviation *λ*_e_/m
1	152.9	0.48	151.1	0.50	151.9	0.46	152.2	0.48
2	156.2	0.45	158.0	0.49	156.7	0.52	157.5	0.45
3	161.6	0.50	160.7	0.55	159.6	0.48	159.9	0.46
mean value of error	2.07	0.03	0.57	0.02	1.43	0.05	1.17	0.05

## Data Availability

Not applicable.
